# Measuring attitudes, behaviours, and influences in inner city victims of interpersonal violence (VIVs) - a Swiss emergency room pilot study

**DOI:** 10.1186/1752-2897-4-8

**Published:** 2010-07-06

**Authors:** Aristomenis K Exadaktylos, Anja Evangelisti, Fiorenzo Anghern, Ursula Keller, Kathrin Dopke, Annette Ringger, Victor Jeger, Heinz Zimmermann, Urs Laffer, Allan Guggenbühl

**Affiliations:** 1 Department of Emergency Medicine, University and University Hospital of Berne (Inselspital), Berne, Switzerland; 2 Department of Emergency Medicine, Biel Hospital Centre AG, Biel, Switzerland; 3 Berne Cantonal Educational Counselling, Berne, Switzerland

## Abstract

**Background:**

Switzerland is confronted with the problem of interpersonal violence. Violence is in the increase and the potential for aggression seems to be rising. Observations by hospitals discern an appalling increase of the severity of the injuries. The aim of this study is to collect accurate information about the social environment, the motivation and possible reasons for violence. We also intend to investigate whether sociocultural, or ethnic differences among male victims exist.

**Materials and methods:**

For the first time in Switzerland, this survey employed a validated questionnaire from the division of violence prevention, Atlanta, Georgia. The first part of the questionnaire addressed social and demographic factors which could influence the risk of violence: age, gender, duration of stay in Switzerland, nationality and educational level. Beside these social structural factors, the questionnaire included questions on experience of violent offences in the past, information about the most recent violent offence and intra and interpersonal facts. The questionnaire itself consists of 27 questions, translated into German and French. In a pilot study, the questionnaire was checked with adolescents for feasibility and comprehensibility.

**Results:**

69 male VIVs were interviewed at two hospitals in the Canton of Bern. Most of the adolescents emphasised that weapons were not used during their confrontations. It is astonishing that all of the young men considered themselves to be victims. Most of the brawls were incited after an exchange of verbal abuse and provocations with unfamiliar individuals. The rivals could neither be classified with the help of ethnic categories nor identifiable groups of the youth scenes. The incidents took place in scenes, where violence was more likely to happen. Interestingly and contrary to a general perception the offenders are well integrated into sport and leisure clubs. A further surprising result of our research is that the attitude towards religion differs between young men with experience of violence and non-violent men.

**Discussion:**

Youth violence is a health issue, which concerns us globally. The human and economic toll of violence on victims and offenders, their families, and on society in general is high. The economic costs associated with violence-related illness and disability is estimated to be millions of Swiss francs each year. Physicians and psychologists are compelled to identify the factors, which cause young people to be violent, to find out which interventions prove to be successful, and to design effective prevention programs. The identification of effective programs depends on the availability of reliable and valid measures to assess changes in violence-related attitudes. In our efforts to create healthier communities, we need to investigate; document and do research on the causes and circumstances of youth violence.

## Introduction

Like other European countries, Switzerland is also struggling with the problem of interpersonal violence [1]. Not only is the extent of the violent offences alarming, is seems that the aggressive potential and the severity of the injuries are increasing too and sadly Switzerland is rapidly approaching European standards [2-10].

Interpersonal violence can be defined as a conscious physical attack, with the intention to cause physical and psychological harm [9,11,12]. Although in Switzerland reports on interpersonal violence appear every day in the tabloid and hospitals treat victims of violence on a regular basis, socio-medical studies on the causes are very rare in our country. The majority of studies concentrate on social factors when discussing the causes and explain interpersonal violence by referring to the "wrong time wrong place" line of argumentation [13-15]. Up until now no studies on the on the social and cultural background of the perpetuators and victims of interpersonal violence (VIVs) have been done in Switzerland. It has to be noted, that as physicians we do not distinguish between attackers and victims. It is not the duty of a physician, to summon the culprits. Therefore, we decided to interview all patients which were involved in and consequently injured in a violent incident. The interviews were done in two hospitals of the Canton of Berne and used a validated U.S. questionnaire [16]. This questionnaire provides researchers and prevention specialists with a set of tools to assess violence-related beliefs, behaviour, and influences, as well as to evaluate programs to prevent youth violence.

Most of the measures in this compendium intend to assess such factors as serious violent and delinquent behaviour, conflict resolution strategies, social and emotional competencies, peer influences, parental monitoring and supervision, family relationships, exposure to violence, collective efficacy, and neighbourhood characteristics. The compendium also contains a number of scales and assessments to measure factors such as aggressive fantasies, beliefs supportive of aggression, attributional biases, and unsocial and aggressive behaviour [16].

Based on our personal experience we hypothesised that VIVs in Switzerland, a country with a very high standard of living and education, may not belong to socially deprived parts of the community, like in other European countries, but can be found within higher educational and social levels too.

Therefore the aim of the study was to collect detailed information about the social environment, the motivation and possible reasons for violent behaviour. We also intended to investigate whether there are sociocultural or ethnic differences among VIVs. To the best of our knowledge, this type of study, with interviews in an emergency shortly after the violent incident, is unique.

## Materials and methods

The University Emergency Department of the Inselspital Berne is the only major emergency department in the city of Berne providing its services 24 hours a day and 7 days a week. Berne is the capital of Switzerland, with over 128,000 inhabitants. The official spoken and written language in Berne is German. 78% of the population of Berne are Swiss nationals, and about 22% hold a foreign passport [2].

The regional hospital of the city of Biel is a Level II emergency department and was chosen as the second study site. Biel/Bienne is officially considered bilingual (french-german), with about 70,000 residents. It is the largest bilingual city in Switzerland. In December 2006, 27% of Biel residents were foreigners [17].

Each of these units sees about 30,000 patients a year,1000 of whom are VIVs.

The first part of the questionnaire addressed social and demographic factors which could influence the risk of violence: age, gender, duration of stay in Switzerland, nationality and educational level. Beside these social structural factors, the questionnaire included questions on experience of violent offences in the past, information about the most recent violent offence and intra and interpersonal facts.

Original Questions selected from the questionnaire.

For the following questions, indicate how many times you did something during the last 7 days.

1. I got angry very easily with someone.

2. I got into a physical fight because I was angry.

3. I slapped or kicked someone.

4. I made someone look stupid with words.

5. I have threatened to hurt or to hit someone.

6. In the past six months, I have attacked or threatened someone with a weapon.

7. In the past six months, I was involved in a gang fight.

8. How often were you involved in a fight in the last twelve months?

9. During the past 12 months, how many times were you in a physical fight in which you were injured and had to be treated by a doctor or nurse? (excluding the current one)?

10. Where did most of your fights take place within the last 12 months?

11. How did the last fight happen?

12. When was the last time that you hurt someone in a fight?

13. What kinds of feelings were caused by this?

14. When was the last time that you have seen a fight without participating in it (excluding the current one)?

15. Who did you fight with most often?

16. Do you have a special type of fighting?

17. Do you attend to be active (participating) or passive (witness) in fights?

18. On which level are most of your confrontations with others?

19. Where I come from is important.

20. I have a clear sense of my religion and what it means for me

21. If I go out, I like to meet people of other nationalities.

22. People of other nationalities make me aggressive and angry.

23. Did you experience violence in childhood?

24. Do you and your environment consume alcohol or drugs before you are involved in fights?

25. Do you identify yourself with an association, a group or a party of which you are a member?

26. What do you do in your free time?

27. For completing this questionnaire, you will receive a CD-coupon amounting to CHF 20. What kind of music are you going to buy with this coupon?

We decided with the help of a psychologist specialized in youth violence (AG) to identify question directly related to hypothesis.

The questionnaire consisted of 27 questions, translated into German and French. In a pilot study, the questionnaire was checked with adolescents for feasibility and comprehensibility.

Included in this prospective survey were VIVs (>16 years) treated at the emergency departments of the two participating hospitals. During the recruitment of the participants, 189 VIVs were approached. The final decision on inclusion was at the discretion of the consultant on call. In total, 100 VIVs agreed to complete the questionnaire, including 69 men and 19 women (mean 22 years, range 16-32 years). The interview costs more time than expected (mean 23 min, range 16-58 min). Therefore the number of patients recruited for this pilot study, has been limited to 100. As a bonus, the interviewed patients received either a CD or a cinema coupon for 20 Swiss Francs (USD 20). This had been found to be useful in previous studies [18-20]. Only the questionnaires of male subjects were evaluated at this stage.

### Data collection

The physicians interviewing the VIVs were trained in advance according to the requirements of the text. After medical treatment, the VIV was asked if he/she was interested in participating. The required coupon was handed over if the patient agreed and filled out the questionnaire. VIVs who could not speak German or French and who were physically and psychologically unable to complete the questionnaire were excluded.

## Results

### General data

48 (70%) of 69 male patients were Swiss citizens. 30% had completed a public school; 35% had finished an apprenticeship; 6% had obtained a university entrance certificate; 4% held a college degree and 1% attended a university.

**Question 1-5**: For the following questions, indicate how many times you did something during the last 7 days.

I got angry very easily with someone.

I got into a physical fight because I was angry.

I slapped or kicked someone.

I made someone look stupid with words.

I have threatened to hurt or to hit someone.

**Figure 1:**
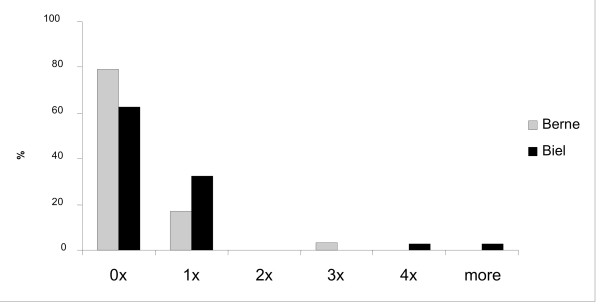
**Fights in the last 7 days**.

**Answers 1-5**: Verbal confrontations and threats occur more often than fights (fig. 1).

**Question 6**: In the past six months, I have attacked or threatened someone with a weapon.

**Figure 2:**
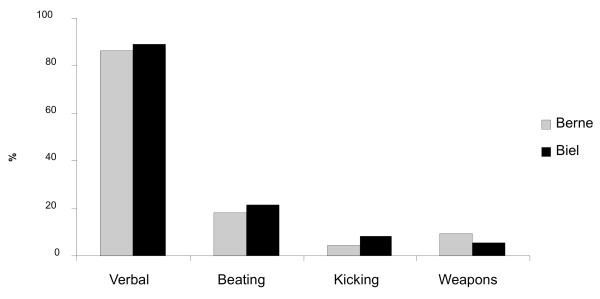
**Levels of confrontation**.

**Answer 6**: Weapons are of minor importance in the Swiss scene (fig. 2).

However, 17% stated that they had participated in a gang fights in the last half year.

**Question 7+8**: In the past six months, I was involved in a gang fight.

How often were you involved in a fight in the last twelve months?

**Answer 7+8**: In the last year, 30% stated that they had been involved in a fight between two to four times, 38% once and 25% never.

**Question 9**: During the past 12 months, how many times were you in a physical fight in which you were injured and had to be treated by a doctor or nurse? (excluding the current one)?

**Figure 3:**
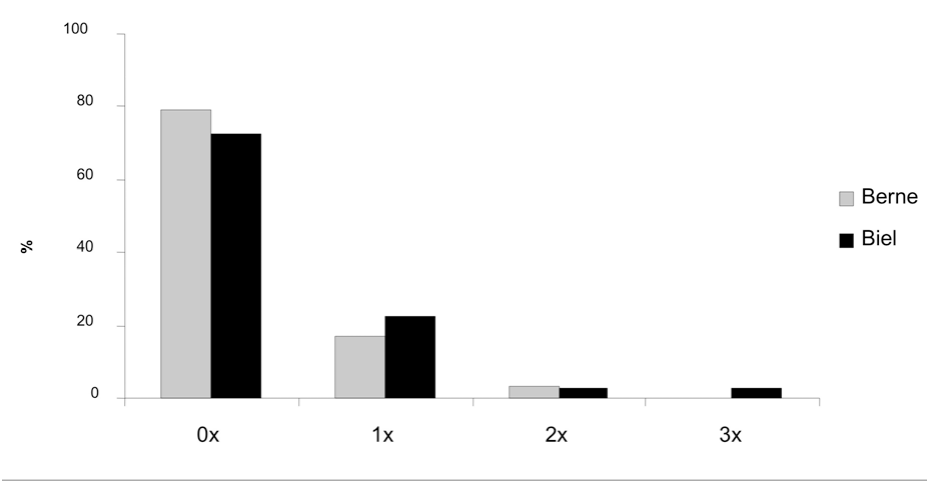
**Injured in a fight in the last twelve months**.

**Answer 9**: 20% of VIVs stated that they had been involved in an additional brawl in the course of the previous year (fig. 3). 76% of the VIVs stated that they had not been involved in any violent confrontation.

**Question 10**: Where did most of your fights take place within the last 12 months?

**Answer 10**: It seems clear that there are no places in Berne or Biel where fights occur with the greatest probability.

**Question 11+12**: How did the last fight happen?

When was the last time that you hurt someone in a fight?

**Answer 11+12**: 26% of the VIVs interviewed had felt provoked; 17% had been verbally insulted and 61% attacked. Interestingly, 62% stated that they had never hurt anyone in a fight. 21% had hurt someone more than one year ago and at least 9% had hurt someone during the last half year.

**Question 13**: What kinds of feelings were caused by this?

**Figure 4:**
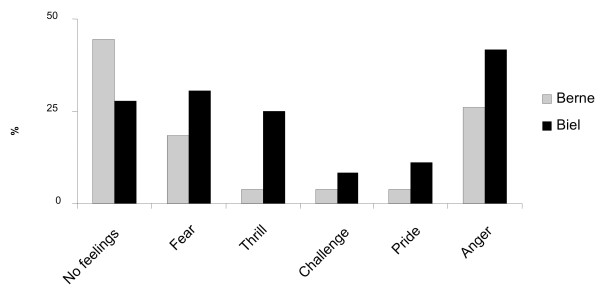
**Feelings during the fight**.

**Answer 13**: 36% asserted that they had had no unusual feelings during the fights, 24% were frightened, 14% experienced a thrill and 34% anger (fig. 4).

**Question 14**: When was the last time that you have seen a fight without participating in it (excluding the current one)?

**Answer 14**: 20% stated that they had witnessed a fight during the last half year and 33% during the last month.

**Question 15**: Who did you fight with most often?

**Figure 5:**
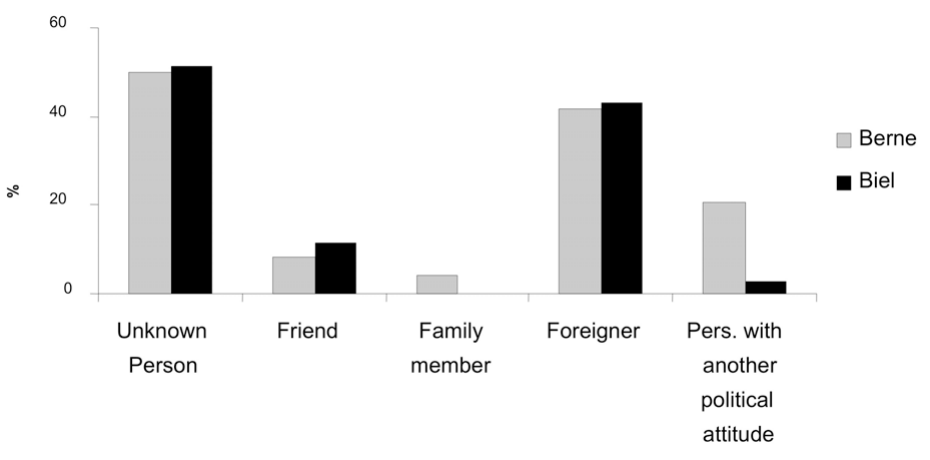
**Opponents during fights**.

**Answer 15**: In most cases, the opponents were strangers (50%); only about 10% were colleagues or adherents of a different political opinion. Family members were not mentioned as adversaries. 42% stated that their opponents were people of other nationalities (fig. 5).

**Question 16**: Do you have a special type of fighting?

**Answer 16**: 17% employed fighting techniques derived from combat sports

**Question 17**: Do you attend to be active (or passive (witness) in fights?

**Answer 17**: 13% asserted that they are active in fights; 50% believed they tended to be passive and for 37% stated this depended on the situation.

**Question 18**: On which level are most of your confrontations with others?

**Answer 18**: Most of the confrontations remained at the verbal level (88%),

**Question 19+20**: Where I come from is important. I have a clear sense of my religion and what it means for me.

**Figure 6:**
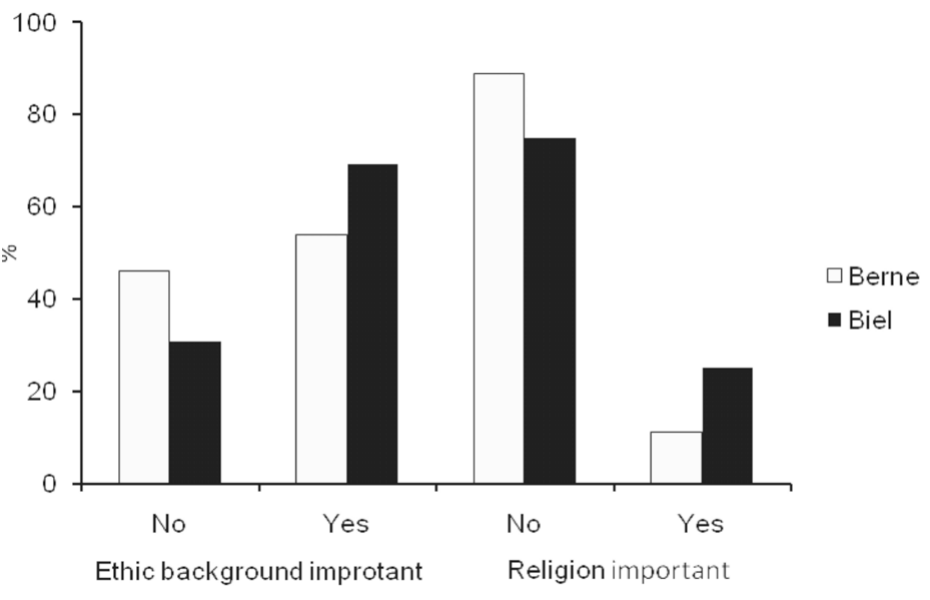
**Importance of origin and religion**.

**Answer 19+20**: The personal origin was important for 39% of the VIVs. 18% stated that their religion was significant for them (fig. 6).

**Question 21+22**: If I go out, I like to meet people of other nationalities.

People of other nationalities make me aggressive and angry.

**Answer 21+22**: 70% stated that they liked to mix socially with foreigners in their free time and 85% stated that they held no grudge against members of other nations.

**Question 23**: Did you experience violence in childhood?

**Figure 7:**
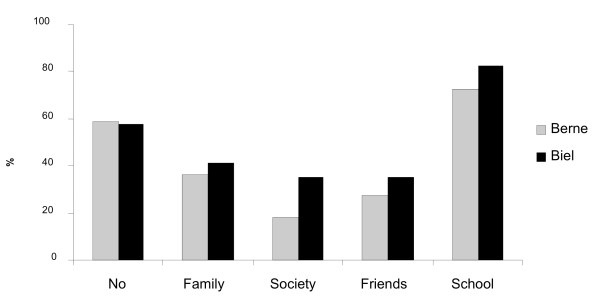
**Experience of violence in childhood**.

**Answer 23**: 41% stated that they had experienced violence during their childhood, 39% in the family and 77% in school. (fig. 7)

**Question 24**: Do you and your environment consume alcohol or drugs before you are involved in fights?

**Answer 24**: 32% had consumed either alcohol or drugs before they became involved in fights.

**Question 25**: Do you identify yourself with an association, a group or a party of which you are a member?

**Answer 25**: Few of the VIVs (31%) considered themselves a member of a distinct group of friends or a sports club (22%).

**Question 26**: What do you do in your free time?

**Answer 26**: The VIVs spent their spare time in many different ways. They had casual meeting with friends (70%), played videogames (25%), played sports (63%) or listened to music (65%).

**Question 27**: For completing this questionnaire, you will receive a CD-coupon amounting to CHF 20. What kind of music are you going to buy with this coupon?

**Answer 27**: Most VIVs were fans of black hip hop or rap music.

## Discussion

Violence among adolescents has become a public concern. Are there any distinct behaviour patterns and personality traits recognisable in VIVs? Do they choose to spend their time in distinct surroundings? Do they pursue specific interests?

### Weapons play a subordinate role

It is interesting that most of the adolescents emphasised that weapons were not used and were of no importance during their confrontations. Instead of guns and knives, they relied on fists. Fortunately for Switzerland, there is no gradual armament of the scene. In contrast to other countries, weapons are still considered a taboo [18]. This is surprising, because Switzerland has relatively slack weapon laws and there is hardly a country in which weapons are so widely distributed and easy attainable [14].

### All of the young men regard themselves as victims

It is astonishing that all young men considered themselves as victims! From their point of view, they were only defending themselves. They seemed convinced, that they had to act, after having been verbally abused, provoked or attacked. They seemed unable to see their part in the incidence. These findings reveal a widely known unconscious defence mechanism. After having been involved in a violent incidence, even the most aggressive perpetuators tend to clear themselves of any personal responsibility. Psychologically they have to see themselves as innocent victims, because any other self-description might arise tension. The vile behaviour creates a conflict with the self-image. Self-images don't contain actual features and character traits, but serve a distinct psychological purpose. Self-images need to be whitewashed from any disturbing features or deeds. Self-descriptions need to be in compliance with the values and attitudes of the social environment we belong to, actions, which contradict these values, must be suppressed. When we reflect our actions, we naturally tune in our habitat. This phenomenon raises several questions about violence prevention. It means, that it is not sufficient to work on the conscious level, when we want to help young people at risk. They might honestly declare, that they abhor violence and believe in peaceful conflict resolutions, while their actual behaviour and reactions tell a different story. In conflict situations unconscious motives prevail. Violent features and thoughts are spontaneously suppressed, because on wants to tune in to a society, which focuses on victims and has great difficulty in accepting violence as a behaviours pattern in ourselves. This means, that prevention work might be of no avail, when we work on a conscious, cognitive level. Promoting peaceful gaols, handing out leaflets against violence or organizing workshops in order to encourage peaceful communication, might not have a big effect. The very first step in violence prevention might be the acceptance of our problematic features. These questions need to be investigated in future studies [8]. How can one tackle violence when there are no perpetrators?

### Injured patients have a violent environment

Most of the fights occurred after verbal provocations from people who were *unfamiliar *and who also could not be classified into an ethnic group or known sociological sub-group. The incidents happened in scenes in which violence is more likely to happen. This indicates, that the social context plays an important role. Violent behaviour is influence by the social surroundings of the young men, might even have certain function with respective social context. These findings are consistent with the existing literature [8].

### Young men who participate in violence are socially integrated

In contrast to a generally held opinion, young men who are engaged in violence participate in sport club. This could be a sign, that they are integrated into society and enjoy acceptable leisure activities. Engaging in sportive activities does not seem to reduce the likelihood of violence. The engagement in sport clubs does not deter young men from being violent. Sportive activities don't seem to be an outlet for aggression either. Our finding also indicate, that violent criminals may not belong to a marginalised stratum and [8].

### Does religion protect against violence?

A further interesting difference between young men with experience of violence and non-violent men is their attitude towards religion. Men without experience of violence value religion more than violent men. Is religion therefore a deterrent? This could mean, that a religious attitude does not foster violence but might even impede it [16]. This finding is difficult to interpret. Maybe young men who are prepared to admit religious thoughts are more aware and self-reflective. It could also be a sign for

### Small group of habitually violent criminals

The data confirms that there is a small group of persistently violent men. According to our data, they have experienced violence during their childhood and adolescence. However, we could not discern whether they were victims or perpetrators [16].

## Limitations

Our conclusions are preliminary, as they have been recorded for a small group of patients. In order to be validated, they have to be confirmed in larger groups of patients. Furthermore: this analysis might be biased because the inclusion of the patients was not consecutive and intoxicated or violent patients were not asked to participate. Some VIVs might have been reluctant to answer sincerely, fearing reprisals or the instigation of interrogations by the police, although they were assured this would not be the case. It is difficult to determine whether the answers are accurate or distorted.

## Conclusion

Youth violence is a serious global public health problem. Despite low violence rates across Switzerland in comparison to other European countries, interpersonal violence is rising and claims the health of many young people. The human and economic toll of violence on victims and offenders, their families, and society in general is high[16]. The economic costs associated with violence-related illness and disability is estimated to be millions of Swiss francs each year. Physicians and psychologists are under pressure to identify the factors that place young people at risk for violence, to find out which interventions are working, and to design more effective prevention programs. Across Switzerland, primary prevention efforts involving families, schools, neighbourhoods, as well as social and ethnic communities appears to be essential to stem the rising tide of violence in the streets of Switzerland. The identification of effective programs rests, in part, on the availability of reliable and valid measures to assess change in violence-related attitudes, beliefs, behaviour, and other influences[16]. Monitoring, documenting, asking and listening will go a long way toward reducing youth violence and creating peaceful, healthier communities.

## Competing interests

The authors declare that they have no competing interests.

## Authors' contributions

AKE has been the principal investigator and drafted the manuscript, AE and FE collected data and interviewed patients, UK reviewed and analysed data, KD coordinated study at both hospital sites, AR supervised study at Biel Hospital, VJ helped revising the manuscript, HZ co-designed study, UL co-designed study, AG designed study and drafted the questions, co-drafted the manuscript. All authors read and approved the final manuscript.
